# Optimal multiobjective design of digital filters using spiral optimization technique

**DOI:** 10.1186/2193-1801-2-461

**Published:** 2013-09-13

**Authors:** Abderrahmane Ouadi, Hamid Bentarzi, Abdelmadjid Recioui

**Affiliations:** Laboratory signals and systems, Institute of Electrical and Electronic Engineering, University M’hamed Bougara Boumerdes, Avenue de l’indépendance, Boumerdes, 35000 Algeria

**Keywords:** Multiobjective filter design, Spiral optimization technique, Magnitude response, Minimum linear phase, Group delay

## Abstract

The multiobjective design of digital filters using spiral optimization technique is considered in this paper. This new optimization tool is a metaheuristic technique inspired by the dynamics of spirals. It is characterized by its robustness, immunity to local optima trapping, relative fast convergence and ease of implementation. The objectives of filter design include matching some desired frequency response while having minimum linear phase; hence, reducing the time response. The results demonstrate that the proposed problem solving approach blended with the use of the spiral optimization technique produced filters which fulfill the desired characteristics and are of practical use.

## Introduction

Digital filters exist in two types: Finite impulse response (FIR) and Infinite impulse response (IIR) or recursive. FIR filters suffer from the problem of high order (hence implementation and performance issues) if strict requirements are imposed at the design stage. Furthermore, IIR filters can have smaller group delay than its equivalent FIR filters (Antoniou 1993 Antoniou 2005). The optimal design of an infinite impulse response (IIR) filter consists in choosing a set of coefficients of the filter to have a frequency response that optimally approximates the desired response (Antoniou 2005; Dumitrescu and Niemisto 2004; Lai 2009; Tseng and Lee 2002 Tseng 2004; Ho et al. 2008; Quelhas and Petraglia 2009; Sanathanan and Koerner 1963).

Different techniques exist for the design of digital filters. Windowing method; in which the ideal impulse response is multiplied by a window function, is the most popular. There are various kinds of window functions (Butterworth, Chebyshev, Kaiser etc.), depending on the requirements on ripples in the passband and stopband, stopband attenuation and the transition width. These various windows limit the infinite length impulse response of ideal filter into a finite window to design an actual response. Furthermore, windowing methods do not allow sufficient control of the frequency response in the various frequency bands and other filter parameters such as transition width. The designer always has to compromise between the design specifications (Antoniou 1993 Antoniou 2005).

Due to the presence of the denominator of the transfer function, the stability condition of the filter should be taken into account in the optimal design (Antoniou 1993 Antoniou 2005; Lu et al. 1998; Saab et al. 1999; Tseng and Lee 2002; Omoifo and Hinamoto 2004; Tseng 2004; Jiang and Kwan 2009 Jiang and Kwan 2010a; Lai and Lin 2010), resulting in a constrained optimization problem. Several sufficient conditions (Lang 2000; Lu 2000; Antoniou 2005; Ho et al. 2008; Pan 2009; Jiang and Kwan 2010b) have been established for the parameterization that represents the filter’s denominator by a single polynomial. The triangle-based stability conditions (Antoniou 1993) are necessary and sufficient and have been incorporated into several design procedures (Lu 1998; Lu and Hinamoto 2003; Lu 2006) that formularize the filter’s denominator by cascaded second-order sections (SOSs). In (Lu 1998), variable transformation is used to convert the finite stability region into the entire coefficient space, such that the original constrained design problem becomes an unconstrained one in the transformed space. However, the transformation increases the nonlinearity of the objective function, which makes it hard to find good (global optimum) solutions in general. In (Lu and Hinamoto 2003), a perturbed stability triangle is proposed to guarantee the SOS to have its zeroes inside a circle of given radius. It is combined with the Gauss–Newton strategy, resulting in an improved design. In (Lang 1999), the conditions presented for the SOS with zeros inside a circle of given radius enclose a triangular stability domain and can be easily incorporated into any constrained optimization formulations based on the SOS parameterization. A method that divides the overall design of an IIR filter into successive designs of its second-order sections is presented in (Saab et al. 1999), where one section is first designed, and then, another section is appended until all sections are designed.

Because of finite word length effects occurring in practical implementations of the designed filters, not only stability of the filter is of great importance but a stability margin is necessary as well. The poles of the transfer function should not lie too close to the unit circle. The sensitivity of pole locations to coefficient quantization increases with decreasing distance from the unit circle. Poles close to the unit circle may considerably enhance quantization noise and increase the maximum amplitude of small scale limit cycle. Consequently, it is desirable to have control over the maximum radius when designing IIR filters.

Linear-phase filters are usually designed as non-recursive (FIR) filters which can have constant group delay over the entire base-band. However, when highly selective filters are required, a very high filter order is needed which makes these filters uneconomical or impractical. To eliminate this problem, attempts have been made to develop methods to design recursive (IIR) filters whose delay characteristics approximate a constant value in the passband. This includes IIR filter design approach that can satisfy both magnitude and phase characteristics simultaneously (Inukai 1980; Cortelazzo and Lightne 1984; Sullivan and Adams 1997; Lang 2000; Lertniphonphun and McClellan 2001). The design of IIR filters with constant group delay in the passband is also carried out by using allpass structures through evaluation of phase response instead of approximating the group delay directly (Jing 1987; Ikehara et al. 1992; Lang and Laakso 1994; Zhang and Iwakura 1999). Some other methods used an indirect approach based on model reduction techniques where a linear-phase FIR filter that meets the required specifications is first designed and then a lower order IIR filter that meets the original amplitude specifications while maintaining a linear-phase response in the passband is obtained (Sreeram and Agathoklis 1992; Peng et al. 1992; Beliczynski et al. 1992). Coretlazzo and Lightner (Coretlazzo and Lightner 1984) have achieved the simultaneous design in both magnitude and group delay of IIR and FIR filters based on multiple criterion optimizations. Lutova (Lutova 1997) has developed a new design method for elliptic IIR filters that provide the implementation of half of the multiplication constants with few shifters and adders. Sullivan et al. (Sullivan James and Adams 1998) have proposed the algorithm based on the peak–constrained least–squares optimality criterion for cascaded IIR filters, which can design a filter that has an equalized group delay without the use of all pass filters, and it can simultaneously meet the frequency response magnitude specifications by using all of the filter coefficients available to optimize the filter. Lang (Lang 2000) has used least square method for designing IIR filter with prescribed magnitude and phase response. This parameterization of the transfer function has been used for designing IIR filters. GordanaJavanovic (Gordana 2006) has proposed a method for the design of IIR notch filters with desired magnitude characteristic, which can be either maximally flat or equi-ripple. Xi Zhang (Zhang 2008) have proposed a novel method for designing maximally flat IIR filters with flat group delay responses in the pass-band.

Under these circumstances, evolutionary and metaheuristic optimization methods find their place. These are referred to as global optimizers while the more familiar, traditional techniques such as conjugate gradient and the quasi-Newtonian methods are classified as local optimizers. The distinction between local and global search of optimization techniques is that the local techniques produce results that are highly dependent on the starting point or initial guess, while the global methods are totally independent of the initial conditions (Recioui 2012). Though they possess the characteristic of being fast in convergence, local techniques, in particular the quasi-Newtonian techniques have direct dependence on the existence of at least the first derivative. In addition, they place constraints on the solution space such as differentiability and continuity; conditions that are hard or even impossible to satisfy in some situations (Recioui 2012).

Previously, global optimization techniques have been implemented in the design of digital filters. One such approach using neural networks has been described in (Wang et al. 2006). Also, use of PSO in the design of frequency sampling finite impulse response (FIR) filter has been described in (Wang et al. 2004; Krusienski and Jenkins 2004; Chen and Chen 2006). Differential evolution has been used in the design of digital filters has been implemented in (Karaboga D 2004; Storn 1996,2005; Karaboga N 2005). (Kit Sang and Kim-Fung 1998; Karaboga N 2004) have used Hierarchical Genetic Algorithms to the design and optimization of IIR filter structures. Use of Particle Swarm Optimization (PSO) and Genetic Algorithms (GA) in the design of digital filters is described in (Ababneh, and Bataineh 2007).

In this work, the application of the novel optimization technique called spiral optimization to the design of digital filters is considered. The purpose is to design a filter that can simultaneously satisfy multiobjective criteria including frequency response and linear phase with the least possible group delay.

## Problem formulation

Digital filters find their applications in different areas. One area is power system protection where measurement systems involve faulted signals associated with DC decaying signals, harmonic and sub-harmonic components. To eliminate these unwanted components, a digital filter design based on multi-objective optimization technique to satisfy different specifications such as high speed response for a real-time application and frequency domain requirements.

### Digital filtering approach

A digital filter based solution is proposed to remove unwanted disturbances using digital filter design techniques. The filter time response must be included in the requirements. The present filtering application imposes different kind of specifications. On one hand, the time domain requirement where both a high speed and accurate system response are needed. On the other hand, the frequency domain requirements (DC, sub-synchronous and harmonic components elimination) which are the magnitude response within small bandwidth including sharp frequency edges as well as an approximately constant group delay in this band are required too. Usually the best optimum value of all the objective functions of this filter design can be obtained for some values of design variables. A compromise or a trade-off between the objective functions must be made to achieve a satisfactory filter design.

The considered recursive digital filter must satisfy three multi-objective functions. These functions are: 1) meet a specified or a desired magnitude response specification; 2) an approximately constant group delay; and 3) a minimum time response or settling time which involves a minimum phase or a group delay. The optimization approach considers the discrete-time transfer function which is formulated on the basis of some desired amplitude response and a stability margin parameter. A norm of the weighted error function is then minimized with respect to the transfer-function coefficients with a prescribed maximum pole radius referred to as stability margin. The stability margin parameter is varied to optimize the filter coefficients which minimizes mainly the magnitude response, satisfies the best approximately constant group delay and the lowest group delay that leads to minimum settling time or time delay of the system dynamic response.

### Filter transfer functions

In the general case an IIR filter can be described by its discrete-time difference equation1

Where x[n] and y[n] are discrete-time input and output signals. Equation (1) can be transformed into the Z-domain and assuming c_i_ and d_i_ are real coefficients a second order form transfer function can be obtained, having 2 M conjugate zeros and 2 N conjugate poles; called second order sections (SOS), as:2

Where a_ii_ and b_jj_ are real coefficients and H_0_ is a positive multiplier constant. The polar formulation is also useful and is written as:3

Where r_aj_, θ_aj_ and r_bj_,θ_bj_ are the radii and angles of the zeros and poles, respectively.

### Filter stability margin

**Figure 1:**
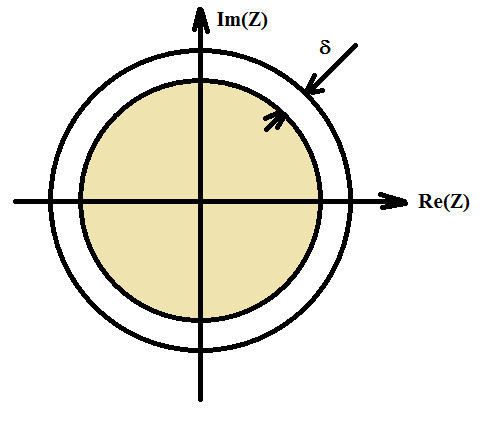
**Stability region and stability margin parameter δ.**

As the poles are moved toward the origin (decreasing the pole radius), the system stability margin parameter increases and the system settling time decreases. This action, in fact, brings two required and important properties to the designed system. First, the system time or dynamic response is enhanced as settling time is decreased. Second, the system stability becomes more robust which is a very useful property, particularly in practical implementation. Indeed, the rounding or truncation of the filter coefficients may lead to an unstable implementation if the stability margin is too small. It is therefore desirable to approximate a given response by a transfer function with a prescribed maximum pole radius named stability margin as shown in Figure 1.

### Magnitude response objective function

The amplitude and the phase responses of a recursive filter is given by4

Where ω is the frequency and x is a column vector with 2 M + 2 N + 1 elements and depend on the used formulation, that is a Cartesian form as5

Or in polar form as6

Where7

And8

The superscript T denotes the transpose operation. An approximation error can be formulated as the difference between the actual amplitude response M(x, *ω*) and the desired amplitude or magnitude response *Μ*_*d*_(*ω*) as:9

By sampling the error function e(x,ω_i_), the actual and the desired amplitude responses M(x,ω_i_) and M_d_(ω) at frequencies: ω_1_, ω_2_, …, ω_k_, the column error vector is constructed as:10

Where *e*_*i*_(*x*) = *M*(*x*, *ω*_*i*_) − *M*_*d*_(*ω*_*i*_) and {*ω*_*i*_ : *i* = 1, 2, ⋯ *k*} is a dense set of frequencies which are distributed over in the pass-band and stop-band of the filter. A weighting or penalty error is included to control portions of the actual filter response curve that are most important to the filter response. This involves modifying the error to the form:11

Where *W*(*ω*) is a weighting piece wise constant function over all frequency space, which is assigned a positive value greater than one or less than one to increase or decrease the magnitude approximation in a given band.

A recursive filter can be designed by finding a point  in (11) such that12

Such a point can be obtained when solving the optimization problem by minimizing the error function . The design of a recursive filter that approaches a specified response *M*_*d*_(*ω*_*i*_), can be performed by minimizing the error objective function in terms of Lp norm error which is formulated as:13

Subject to *r*_*aj*_ ≤  1 − *δ*

Where14

And

The p is a positive integer. The spiral technique is used to minimize *μ*(*x*) for increasing values of p.

### Group-delay objective function

The group delay is derived from the phase relation, as given in equation (4), and is defined as:15

Where *φ*(*x*, *ω*) is the phase response of the filter, and stated as16

The group delay for a synthesized recursive filter is desired to be unchanged in the considered region on one hand. On the other hand, a minimum group delay is another required property needed in certain applications. In the present design, a constant group delay property is considered which is defined as:17

Where *τ*_*c*_ is constant, and *ω*_*p*_ is the passband regions of the filter. In the present application *τ*_*c*_ is an unknown but can be considered as the mean value over the passband region, which can be determined as:18

Where  is the optimal filter coefficient determined by minimizing the magnitude objective function for an m-th stability margin parameter.

The stability margin parameter is varied for discrete values, from which an optimal constant group delay is determined by minimizing the following objective error function:19

The multi-objective optimization problem is solved by discretizing the stability margin parameter, the magnitude optimization algorithm is used to generate the corresponding filter coefficients’, in which basis the group delay is synthesized where a feasible and optimal solution can be obtained by minimizing the objective function (19).

The final multiobjective design is obtained by minimizing the sum combination of the errors in equations (14) and (19).

## The spiral inspired optimization method

Compared with traditional optimization techniques and other global optimizers, the spiral optimization method is easy to implement and very efficient in reaching optimum solutions. Spiral optimization method has been recently developed based on the analogy to spiral phenomena (Tamura and Yasuda 2011a; Tamura and Yasuda 2011b).

Patterns in nature are visible regularities of form found in the natural world. These patterns recur in different contexts and can sometimes be modelled mathematically. Natural patterns include symmetries, trees, spirals, meanders, waves, foams, arrays, cracks and stripes. Mathematics, physics and chemistry can explain patterns in nature at different levels. Patterns in living things are explained by the biological processes of natural selection and sexual selection. Studies of pattern formation make use of computer models to simulate a wide range of patterns.

**Figure 2:**
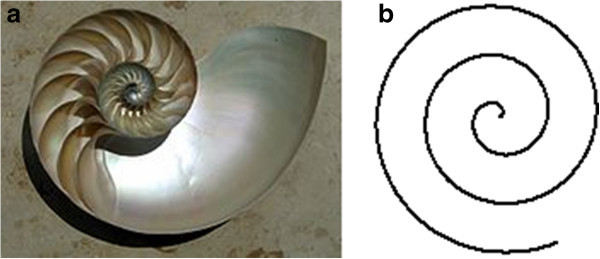
**Natural spiral patterns and their mathematical model.(a)** Cutaway of a nautilus shell. **(b)** Logarithmic spiral.

Among the natural patterns, spirals are common in plants and in some animals. For example, in the nautilus (Figure 2a), each chamber of its shell is an approximate copy of the next one, scaled by a constant factor and arranged in a logarithmic spiral.

The spiral phenomena occurring in nature (like the one in Figure 2a) are approximated to logarithmic spirals as in Figure 2b. Examples of natural spiral dynamics include whirling currents, low pressure fonts, nautilus shells and arms of spiral galaxies. Logarithmic spirals discrete processes to generate spirals that can form an effective behaviour in metaheuristics. A two-dimensional algorithm has been first proposed (Tamura and Yasuda 2011a), and then, a more generalized n-dimensional version has been recently suggested (Tamura and Yasuda 2011b).

In the present work, the use of the spiral optimization technique is presented and used to solve the multiobjective IIR filter design. First, based on practical requirements involved in power system application, a detailed mathematical IIR filter design formulation is presented.

Next, the spiral optimization algorithm detailed and implemented to solve the optimization task. A first optimization example aims at matching a desired magnitude response only. Later, the design is improved by considering the other requirements including minimum and constant group delay.

Before presenting the n-dimensional spiral optimization algorithm, it is worth understanding the two dimensional optimization model as some results are just extended over.

### Two-dimensional spiral optimization

**Figure 3:**
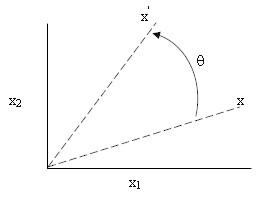
**Rotation in ×**_**1**_**-×**_**2**_**plane.**

Rotating a point in a 2-dimensional orthogonal coordinate system (as shown in Figure 3) to the left around the origin by *θ* can be expressed as:20

Where21

Hence, the two dimensional algorithm moves from one point to another as:22

Where *θ* is the rotation angle around the origin (0 ≤ *θ* ≤ 2*π*) and *r* is the convergence rate of distance between a point and the origin at each iteration *k* (0 < *r* < 1). The parameter r can be regarded as the “scaling” or radius of the logarithmic spiral curvature.

The spiral model presented earlier has a center only at the origin. Hence, it should be extended to have center at an arbitrary point x* as:23

Based on the previous formulation, the following optimization algorithm may be developed:

- ♦ **Preparation:** select the number of search points m > 2, the parameters *θ* and *r* and the maximum number of iterations *k*_*max*_.
- ♦ **Initialization:** initialize randomly the points; *x*_*i*_(0) *i* = 1..m; in the feasible region and the center x* as the point with the least fitness value.
- ♦ **Updating***x*_*i*_:24
- ♦ **Updating**x*: Select x* as the point with the least fitness function in the updated set of points.
- ♦ **Check for termination criterion:** If *k* = *k*_*max*_ then stop. Otherwise, start a new iteration.

### n-dimensional spiral optimization

The extension of the two-dimensional optimization algorithm presented earlier is easy to do as one must understand how rotation in an n-dimensional space is done. Rotation in n-dimension is performed in the same way as the two-dimensional rotation taking two dimensions at a time. This is defined for dimensions *i*, *j* as:25

Where the blank elements are zeros.

Hence, there are  rotation matrices. The resulting rotation matrix is then (Tamura and Yasuda 2011b):26

Hence the n-dimensional algorithm may be formulated similar to the two-dimensional algorithm as:

- ♦ **Preparation:** select the number of search points m > 2, the parameters *θ* and *r* and the maximum number of iterations *k*_*max*_.
- ♦ **Initialization:** initialize randomly the points; *x*_*i*_(0) *i* = 1..m; in the feasible region and the center x* as the point with the least fitness value.
- ♦ **Updating***x*_*i*_:27
- ♦ **Updating:**x*: Select x* as the point with the least fitness function in the updated set of points.
- ♦ **Check for termination criterion:** If *k* = *k*_*max*_ then stop. Otherwise, start a new iteration.

**Figure 4:**
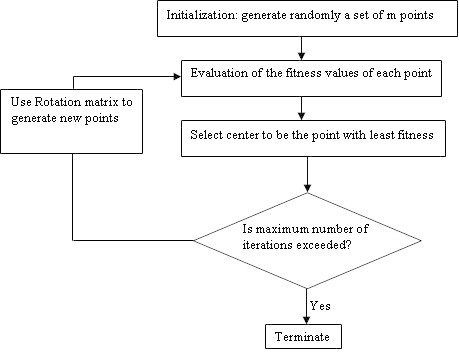
**The spiral optimization algorithm flowchart.**

The flowchart in Figure 4 summarizes the spiral optimization procedure.

## Results and discussions

The digital filter to be optimized is to be used to eliminate harmonics and sub-harmonics in a power network with a fundamental frequency of 50 Hz. The filter is a bandpass type and is desired to satisfy the magnitude response to ideally pass only frequencies confined in the interval [45 Hz, 55 Hz] while rejecting all other frequency content. The sampling frequency is taken to be 1800 Hz. The filter is of order 10 and is hence composed of 5 cascaded SOSs. At start up, the filter is optimized to match the magnitude response specifications only. Next, more constraints are added to the optimization process including minimum and linear phase and constant group delay to enhance the designed filter performance.

### Single objective design

**Figure 5:**
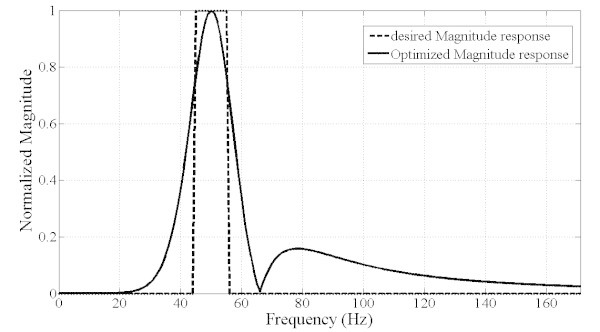
**Magnitude filter response (SOOF).**

The purpose of this part is to design a filter which only satisfies the magnitude response described earlier without considering any other performance criteria. The filter is designated single objective optimized filter (SOOF) hereafter. Figure 5 shows both the desired and the optimized magnitude response of the digital filter. It can be noted that the filter fulfils well the requirements of magnitude response. Indeed, the filter response falls exactly within the desired frequency range [45 Hz, 55 Hz] and it attenuates all other frequencies as the overall Sidelobe level is lower than −16 dB.

**Figure 6:**
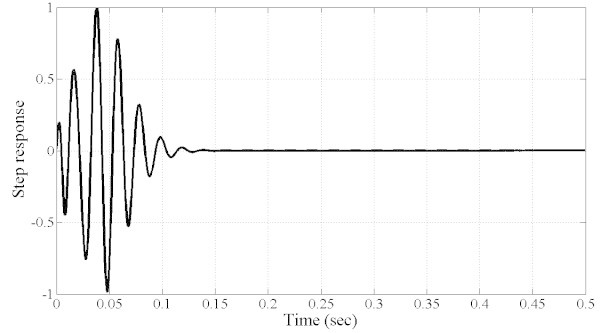
**Step response of the filter and time delay (about 100 ms) (SOOF).**

**Figure 7:**
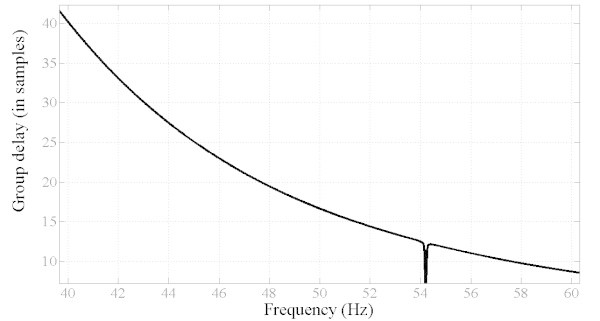
**Goup delay response of single objective optimized filter (SOOF).**

**Figure 8:**
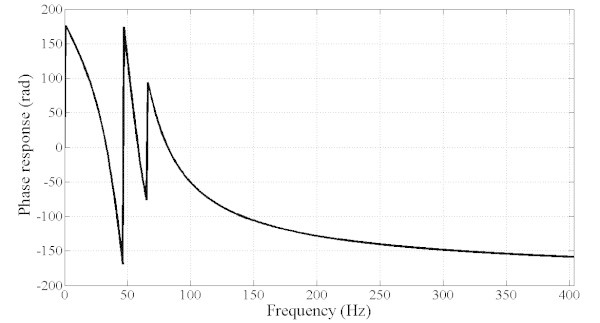
**Phase response of filter with non linear phase or non constant group delay (SOOF).**

However, the optimized filter is not of practical use as it suffers from drawbacks in the dynamic properties. First, as shown in Figure 6, the time delay is in the order of 100 ms which is not suitable in the present applications as the requirements specify that the time delay should not exceed one cycle (20 ms). In addition, the group delay is not constant as shown in Figure 7. Furthermore, the phase response is nonlinear as shown in Figure 8 which explains the non constant nature of the group delay. As a result, it is necessary to include all the preceding performance criteria into the design process and hence, the problem becomes a multiobjective optimization task. The results are summarized in Table 1 where are presented the filter SOS coefficients and SOS gains.

**Table 1:** **Single-objective optimized filter (SOOF) SOS coefficients and gains**

Section	Numerator			Denominator			Gain
**1**	1	−1,9520335260	0,9999998000	1	−1,8473812327	0,8836	0,00089772300915
**2**	1	−1,9999996000	0,99999980000	1	−1,9999996000	0,999999800000010	1
**3**	1	−1,9999996000	0,99999980000	1	−1,8473812327	0,88360000000	1
**4**	1	−1,8851094580	0,99999980000	1	−1,8473812327	0,883600000000	1
**5**	1	1,99999980000	0,99999980000	1	−1,8473812327	0,88360000000	1

### Multiobjective filter design

**Figure 9:**
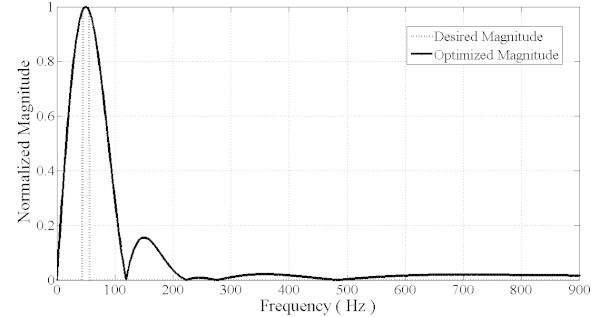
**Magnitude filter response (MOOF).**

The inclusion of the constant and minimum group delay in the optimization task besides magnitude response criterion produced a filter which satisfies almost all requirements. The filter is thereafter called multiobjective optimized filter (MOOF). The magnitude response of the MOOF is shown in Figure 9. The filter magnitude response is not as good as the magnitude response characteristic of the SOOF. Indeed, the SOOF bandwidth is narrower than the MOOF which makes it having better selectivity.

**Figure 10:**
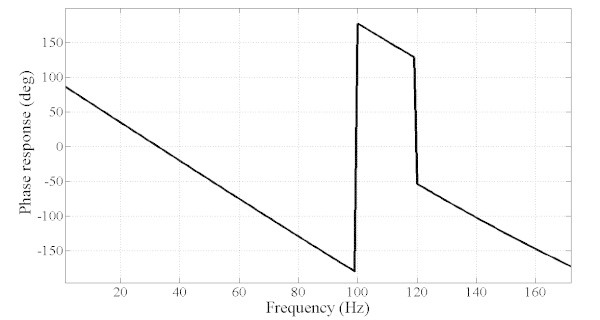
**Optimized filter phase response MOOF.**

**Figure 11:**
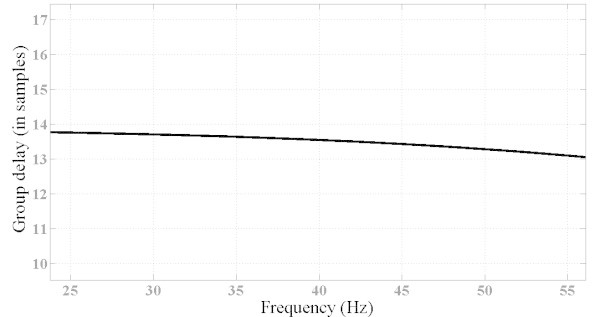
**Group delay response of optimized filter MOOF.**

**Figure 12:**
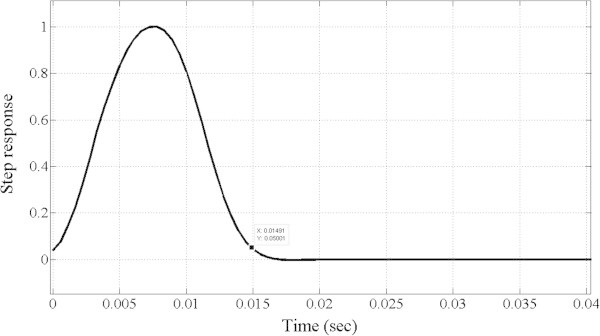
**Optimized filter step response and time delay (14.9 ms) MOOF.**

However, the MOOF performs well in terms of the other criteria. In terms of phase response and group delay, Figures 10 and 11 illustrate the phase and group delay responses of the MOOF. The filter is characterized by a linear phase with an almost constant group delay in the pass-band contrary to the SOOF phase response and group delay illustrated in Figures 7 and 8. Furthermore, the group delay is low yielding a small time response as shown in Figure 12. Indeed, the time delay of the MOOF is about 14.9 ms which conforms to the desired specifications. This is due to the fact that the stability margin or equivalently the pole radii have been taken into account in the optimization process and these latter have been lowered to a value of 0.595 instead of being closer to unity. Table 2 summarizes the SOS coefficients and gains of the MOOF.

**Table 2:** **Multi-objective optimized filter (MOOF) SOS coefficients and gains**

Section	Numerator			Denominator			Gain
**1**	1	−1,82933833319	0,999999800000	1	−1,0914383222	0,35402500000	0,012499922808
**2**	1	−1,4247565639	0,99999980000	1	−1,0914383222	0,35402500000	1
**3**	1	−1,1469437066	0,99999980000	1	−1,1899988100	0,35402500000	1
**4**	1	0,227981442276607	0,999999800000010	1	−1,09143832226114	0,354025000000000	1
**5**	1	−0,467539619942456	−0,532460226811516	1	−1,11373644574629	0,354025000000000	1

To validate the obtained results, the MOOF optimized filter is compared with the classical filter designs widely used in literature. The filters have the same order and should meet the same requirements described earlier. The filters considered are the least p^th^ optimized filter, the elliptic and Chebyshev type II filters.

**Figure 13:**
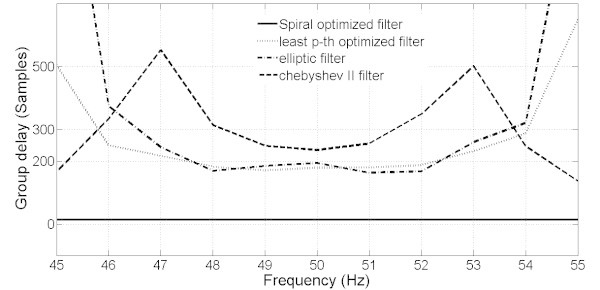
**Group delay comparison between the MOOF and the classical filter designs.**

The classical filters have magnitude responses that meet well the requirements as compared to the MOOF. This can be explained by the fact that these filters are mainly designed to match a given magnitude response which can be considered to be similar to the SOOF optimized filter. However, according to Figures 11 and 13 the MOOF has lower and constant group delay over the pass-band region compared to the classical filters. In fact, the delays of the classical filters are in orders of 200 and 300 sample delay (the MOOF delay is at 14 samples) which is not acceptable for practical requirements. Also, the classical design approach relies on matching a required magnitude response followed by an all-pass filter equalizer. Hence, the classical design adds on the system order and the computational complexity. The latter approach is not suitable for real time applications.

**Figure 14:**
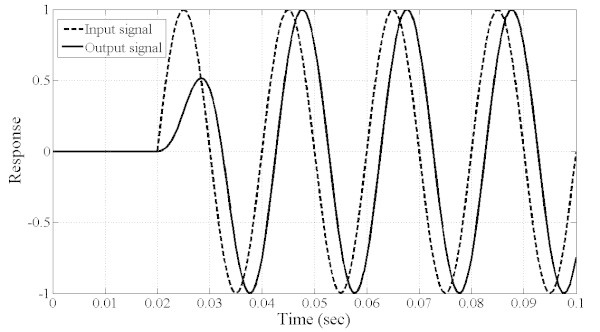
**Test signal: step of sinusoidal signal input and filter output response.**

**Figure 15:**
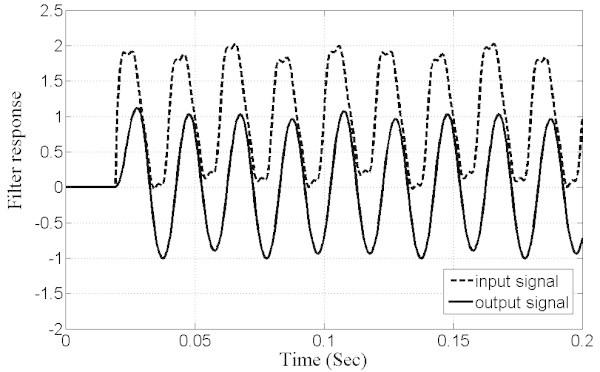
**Second test signal by considering a step sinusoidal signal mixed with harmonic and subharmonic components.**

To better assess the performance of the filter (MOOF), two tests have been performed. In the first, a step sinusoidal signal of 50 Hz is input to the filter and Figure 14 shows both filter input and output waveforms. It is seen that the filter output matches exactly the input except for a phase shift and a time delay of less than one cycle. Hence, this filter proves to be practical for high speed measurement systems where the system accuracy is of great importance. In the second test, the previous step sinusoidal signal is corrupted with a DC offset, harmonic and subharmonic components. The subharmonic component is set to 25 Hz and the harmonics to 100, 150 and 200 Hz. Both input and output signals are shown in Figure 15. As it is clearly seen, the filter succeeded in eliminating the DC and harmonic components and mitigating the subharmonic component.

## Conclusion

The application of the spiral optimization method to design a multiobjective digital filter has been considered in this paper. The objectives of the filter design were to match a desired magnitude response while having a minimum and linear phase. At start up, only magnitude response has been considered in the optimization task. The resulting filter was good in terms of this characteristic while it showed awful dynamic and phase performance. Next, the dynamic properties were included in the optimization algorithm to solve a multiobjective task. The spiral optimization method has succeeded in attaining the optimal design in terms of the previous requirements by achieving a compromise between them. The optimized filter has been tested and it showed good performance with required practical characteristics.
